# 1839. Social Determinants of Health in Extended Spectrum Beta-Lactamase Carriage in the Urine – The Influence of Race, Ethnicity and Sex in the Outpatient Setting

**DOI:** 10.1093/ofid/ofad500.1668

**Published:** 2023-11-27

**Authors:** Jenna Herkness, Matthew Sims

**Affiliations:** Corewell Health, Hazel Park, Michigan; William Beaumont University Hospital, Corewell Health, Royal Oak, Michigan

## Abstract

**Background:**

Urinary tract infections while common in the general population are frequently overdiagnosed in patients with asymptomatic bacteriuria. Overtreatment of asymptomatic bacteriuria leads to increasing levels of antibiotic resistance. Demographic factors including age and sex are associated with increased antibiotic resistance. There has been little to no prior research on the association of race or ethnicity with antibiotic resistance. Here we report the results of a retrospective analysis of urine cultures and the associations of race, ethnicity, age, and sex with the presence of extended spectrum beta-lactamases (ESBLs).

**Methods:**

All urine cultures within the electronic medical record of Corewell Health East were screened for adult outpatients who live in the 3 counties within the main catchment area in southeast Michigan (Wayne, Macomb, and Oakland counties) with positive urine cultures for the year 2019. We identified 22,169 positive urine cultures, of which 1,571 were ESBLs. Demographics for each patient with an ESBL were analyzed including race, ethnicity, sex, age, and zip code. Zip codes were used to determine median annual household income using the 2020 United States Census records and 2019 Internal Revenue Service reports. Logistic regression estimated the association between race, ethnicity, sex, age, and income and presence of an ESBL in the urine. A stepwise regression was also performed to determine the strongest predictors of ESBL.

**Results:**

Asian race (OR=3.06, 95% CI 2.41 – 3.89), Arabic/Middle Eastern ethnicity (OR= 2.48, 95% CI 1.99 – 3.09) and male sex (OR=2.02, 95% CI 1.76-2.31) were associated with ESBLs. Age showed an increase in the odds ratio of 1.014 for every year over the age of 18 (95% CI 1.012-1.017). No association was found with median annual household income and ESBL.

**Conclusion:**

These findings show a clear relationship between race, ethnicity, sex, and age and ESBL which appears to be independent of economic status. This serves as proof of concept that social determinants of health are associated with antibiotic resistance. Future work should expand on these relationships in other types of infections which can then be utilized in predictive models of antibiotic resistance geared at improving empiric antibiotic selection in diverse populations.

**Disclosures:**

**Matthew Sims, MD, PhD**, Astra-Zeneca: Investigator for company-sponsored studies|ContraFect: Investigator for company-sponsored studies|Crestone: Investigator for company-sponsored studies|Finch: Investigator for company-sponsored studies|Janssen: Investigator for company-sponsored studies|Leonard-Meron: Investigator for company-sponsored studies|Merck and Co: Investigator for company-sponsored studies|OpGen Inc: Advisor/Consultant|OpGen Inc: Investigator for company-sponsored studies|Pfizer: Investigator for company-sponsored studies|Prenosis: Advisor/Consultant|Prenosis: Investigator for company-sponsored studies|QIAGEN Sciences LLC: Investigator for company-sponsored studies|Roche: Investigator for company-sponsored studies|Seres Therapeutics: Investigator for company-sponsored studies

